# The Epidemic of Peripheral Neuritis

**Published:** 1900-12-15

**Authors:** 


### THE EPIDEMIC OF PERIPHERAL NEURITIS.

THE chief point of interest which has been recorded lately about the epidemic of peripheral neuritis in Manchester and the northern districts is the statement made by Dr. Abram[1] and Dr. Nathan Raw that they have found arsenic in the urine of patients suffering from this disease. The matter is of some importance in reference to the suggestion which has been made by some observers that the symptoms which have been so generally attributed to arsenic may be due to beri-beri, a disease which is unfamiliar to the great majority of English practitioners for, although many of the symptoms complained of in this outbreak bear a strong resemblance to those of beri-beri, the discovery of arsenic in the urine of the sufferers goes far to prove that this poison is the real cause of the mischief. There is, however, another side to this question. It must be remembered that our knowledge of the etiology of beri-beri is even less complete than has been our knowledge of the vagaries of arsenical poisoning, and Dr. Ross,[2] of Liverpool, admitting the extraordinary similarity of some of the cases of peripheral neuritis to certain phases of beri-beri, suggests the question whether this disease, beri-beri, may not itself be due to chronic arsenical poisoning. After examining some of the cases which have occurred at Chester, he says that the symptoms were so suggestive that, had they been met with in a beri-beri region, he thinks there are few medical men who would not have returned them under that heading. Assuming these cases, then, to be really due to arsenical poisoning, he asks whether similar epidemics occurring in the East or on board ships may not often have been wrongly attributed to beri-beri. The possibilities of arsenical poisoning among the natives of Burma and India, and in gaols and asylums, are manifold. Again the sudden disappearance of beri-beri from the Japanese navy after a change of diet is very suggestive, while the sudden improvement which takes place in most cases of beri-beri after their removal from the house where they contracted the malady, is certainly far more suggestive of intoxication than of infection. It has long been thought on epidemiological grounds that beri-beri may be due to some form of food poisoning; it now seems just possible that the poison may be, at least in some cases, nothing but arsenic. Among what may be termed the curiosities of the present epidemic is a report by Dr. Brown,[1] of Bacup, of a case of poisoning by arsenical beer in a child aged two years. In this case there was tenderness of the feet, the face was flushed, the eyes were watery, and there was some slight running from the nose. Erythematous areas were present over the arms and forearms, and in the region of the elbows, most marked on the extensor surfaces. The palms were red, especially over the thenar and hupothenar eminences and about the tips of the fingers. There was also erythema over both knees, and there were several distinct brownish patches of pigmentation on the lower extremities. The father of this child keeps a public house, and the child, although so young, was accustomed to get little "sups" of beer from the customers. Samples of this beer were found on examination to contain traces of arsenic.

[1] Brit. Med. Jour., Dec. 8. [2] Lancet, Dec. 8.

